# 14-3-3 phosphoprotein interaction networks – does isoform diversity present functional interaction specification?

**DOI:** 10.3389/fpls.2012.00190

**Published:** 2012-08-20

**Authors:** Anna-Lisa Paul, Fiona C. Denison, Eric R. Schultz, Agata K. Zupanska, Robert J. Ferl

**Affiliations:** ^1^Program in Plant Molecular and Cellular Biology, Horticultural Science Department, University of FloridaGainesville, FL, USA; ^2^Interdisciplinary Center for Biotechnology Research, University of FloridaGainesville, FL, USA

**Keywords:** Arabidopsis, GRF, plant, subcellular localization, 14-3-3 isoform specificity

## Abstract

The 14-3-3 proteins have emerged as major phosphoprotein interaction proteins and thereby constitute a key node in the Arabidopsis Interactome Map, a node through which a large number of important signals pass. Throughout their history of discovery and description, the 14-3-3s have been described as protein families and there has been some evidence that the different 14-3-3 family members within any organism might carry isoform-specific functions. However, there has also been evidence for redundancy of 14-3-3 function, suggesting that the perceived 14-3-3 diversity may be the accumulation of neutral mutations over evolutionary time and as some 14-3-3 genes develop tissue or organ-specific expression. This situation has led to a currently unresolved question – does 14-3-3 isoform sequence diversity indicate functional diversity at the biochemical or cellular level? We discuss here some of the key observations on both sides of the resulting debate, and present a set of contrastable observations to address the theory functional diversity does exist among 14-3-3 isoforms. The resulting model suggests strongly that there are indeed functional specificities in the 14-3-3s of Arabidopsis. The model further suggests that 14-3-3 diversity and specificity should enter into the discussion of 14-3-3 roles in signal transduction and be directly approached in 14-3-3 experimentation. It is hoped that future studies involving 14-3-3s will continue to address specificity in experimental design and analysis.

## INTRODUCTION

The 14-3-3s are a family of regulatory proteins that is present in all eukaryotes and involved in protein interactions mediating signal transduction pathways. Numerous pathways and processes rely on the 14-3-3 interactions to regulate key metabolic points (e.g., Aitken et al., 1992; Ferl, 1996; Finnie et al., 1999; Fulgosi et al., 2002). The conservation of 14-3-3 structure among eukaryotes speaks to their essential nature, yet the divergence into so many isoforms in each of these species suggests an intricate network of roles for these proteins (e.g., Rosenquist et al., 2000; Roberts and de Bruxelles, 2002; Sehnke et al., 2002b). Most plants have about a dozen 14-3-3 genes that provide sequence and functional diversity potentially leading to specialized structures and functions within the various members of the 14-3-3 family of proteins. That diversity can be enlarged by selective phosphorylation of 14-3-3s at several known sites across the protein, sites that are variously retained among the isoforms. The purpose of this paper is to examine the evolutionary and biochemical diversity of 14-3-3s and question whether this diversity has any intrinsic biochemical, cellular, or physiological significance; then present approaches to answer that question.

14-3-3s are by far the major, most numerous phosphoprotein interaction proteins in plants (e.g., Ferl, 1996, 2004; Ferl et al., 2002; Chevalier et al., 2009; Gokirmak et al., 2010; Denison et al., 2011). This fact alone puts 14-3-3s among the most important interaction nodes in the Arabidopsis interactome (Braun et al., 2011). The 14-3-3s interact with phosphorylated peptide sequences through sequence-specific motifs RSxpSxP, RSxxpSxP, and YpT, among others (Aitken et al., 1992; Dubois et al., 1997a; Yaffe et al., 1997; Johnson et al., 2001; Ferl et al., 2002; Shen et al., 2003). The phosphorylated serine or threonine forms both hydrogen and ionic bonds with highly conserved residues found in the basic binding pocket of the 14-3-3 protein. In vertebrate isoform 14-3-3τ, these residues are located at K49, R56, and R127 (Muslin et al., 1996). Binding can also occur with non-phosphorylated sequences, such as GHSL (Andrews et al., 1998) and WLDLE (Petosa et al., 1998). These diverse binding sequences permit the 14-3-3 proteins to participate in a wide range of phosphorylation-based signaling pathways. Some examples in plants include red light signaling, immunity-associated cell death, abiotic stress response, ATP synthase activity in both mitochondria and chloroplasts, and nitrate reductase activity regulation (Ferl et al., 1994; Jarillo et al., 1994; Bunney et al., 2001; Pozuelo et al., 2001; Folta et al., 2008; Oh et al., 2010).

**FIGURE 1:**
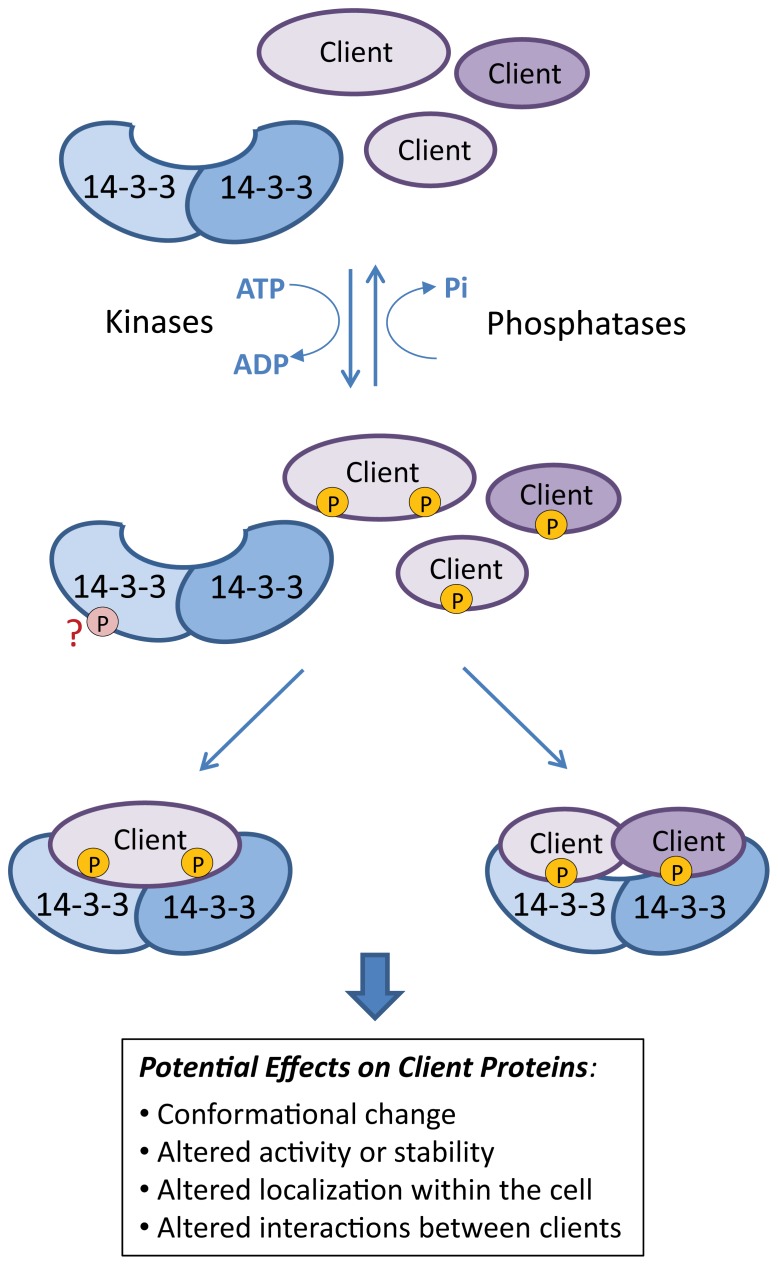
**Function of 14-3-3s.** The basic 14-3-3-mediated regulation of client activity is a two-step process whereby the target protein is first phosphorylated by a specific kinase. This creates a recognition site for the binding of dimeric 14-3-3 proteins, which can have a number of different effects on client activity. Each 14-3-3 monomer can bind to different sites on the same client protein or each can bind different client proteins. 14-3-3s may themselves be phosphorylated (as indicated by “?”) although the effects on client binding are not well understood.

We consider that, in many signaling systems,14-3-3s serve to complete the phosphorylation-based, signal-induced changes in their client proteins, and that the binding of 14-3-3s finalize the signaling event by enabling a change in client conformation, activity, localization, or the association of the client within a larger protein complex (Sehnke et al., 2002a; **Figure 1**). This consideration, that 14-3-3s should serve major phosphor-signaling roles, helps set the stage for the major hypothesis under discussion in this paper. With so many potential roles and many 14-3-3 family members what range of specificities among family members might be expected with regard to biological functions of 14-3-3s?

### 14-3-3 PRIMARY SEQUENCE EVOLUTION

**FIGURE 2:**
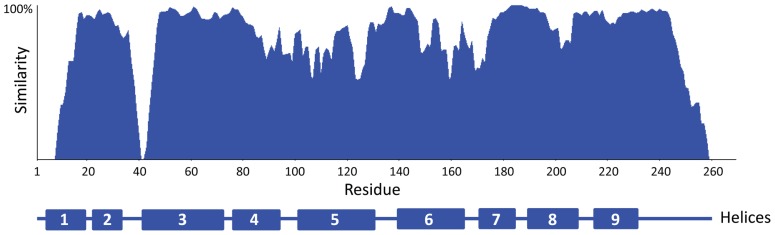
**The degree of amino acid similarity across all 13 Arabidopsis 14-3-3s is displayed graphically.** Amino acid sequences were aligned in Vector NTI and generated with a resolution window of five residues. The nine α-helices are shown below (after Sehnke et al., 2002a).

The 14-3-3s are considered to be a well conserved protein family, yet some areas of the proteins have undergone diversification. The number of isoforms present differs among species. Thirteen expressed isoforms are present in Arabidopsis, seven are present in many animals, and two are present in yeast (Ferl, 1996; van Heusden et al., 1996; DeLille et al., 2001; Aitken, 2006; Schoonheim et al., 2007; Li and Dhaubhadel, 2011). Amino acid identity is conserved to a reasonable degree within the entire family across species and across most of the protein (Comparot et al., 2003; Bridges and Moorhead, 2005; Lottersberger et al., 2006; Zannis-Hadjopoulos et al., 2008; see **Figure 2**).

**FIGURE 3:**
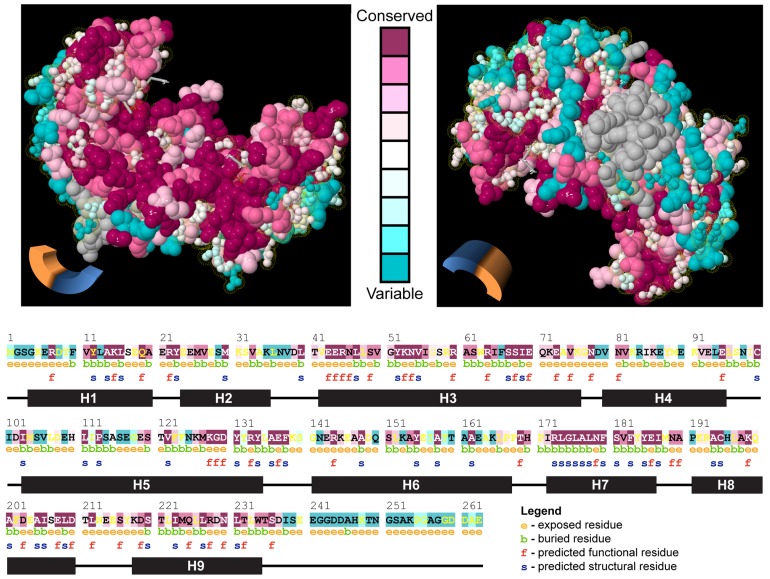
**Amino acid conservation among the 13 Arabidopsis 14-3-3 isoforms.** The Amino acid conservation among the 13 Arabidopsis 14-3-3 isoforms is mapped onto the 14-3-3 protein structure (based on that of human 14-3-3τ/θ) using ConSurf (Landau et al., 2005; Ashkenazy et al., 2010; top panel). Color-coding indicates the amount of change each position has undergone over evolutionary time. Views are angled with perspectives shown in lower left corners, with monomers in orange and blue. Residue conservation is highest in the client binding channel, which is exposed in the left image. The consensus 14-3-3 amino acid sequence is color-coded according to the degree of conservation determined with ConSurf (bottom panel). Residues predicted to have roles in 14-3-3 structure (conserved and buried) and function (conserved and exposed) are annotated below the sequence. Exposed and buried residues were determined using a neural network algorithm. Residues for which there was insufficient data (present in less than 10% of isoforms) are indicated in yellow. Positions of the nine alpha-helices are indicated below the sequence.

Sequence alignment of the thirteen 14-3-3 proteins from Arabidopsis allows the visualization of regions of that have been conserved or have diverged over evolutionary time. These regions can also be overlain onto a structural model to help predict regions that may be important structurally or functionally (**Figure 3**). Although there are regions that show high conservation across all isoforms, it is apparent that there are certain sub-groupings of isoforms that show greater sequence similarity to each other. Conservation patterns can be logically classified along well-supported evolutionary lineages which may imply a specialization-driven divergence (Ferl et al., 1994). This potentially suggests that selection pressures are present in the family due to their involvement in specialized function and complex formation (Rosenquist et al., 2001), but this is a suggestion based on divergence branching pattern and not on the acquisition or loss of known functionalities. Plant 14-3-3s can be divided into two main subgroups, the ε and non-ε groups (DeLille et al., 2001; Sehnke et al., 2002b; Yao et al., 2007; Li and Dhaubhadel, 2011). The non-ε group can be further subdivided, with four of these groupings occurring in the last 170 million years (Piotrowski and Oecking, 1998; Vision et al., 2000; Rosenquist et al., 2001). Additionally, the ε group tends to have deep branching in all plant species (Piotrowski and Oecking, 1998), suggesting that ε isoforms retain ancestral protein function (Wang and Shakes, 1996). The presence of multiple isoforms, however, leads to the question of functional differences. Are these different isoforms performing different cellular functions? Or do these proteins act redundantly as backups to perform these vital functions?

Specificity among 14-3-3s does not appear to be associated with phylogenetic speciation. After consideration of 14-3-3 evolution in plants, there emerges no compelling case for specificity that is based on evolutionary lineages alone. There has been no evolutionary premise that has led to the discovery of specific biochemical interaction or cellular function among plant 14-3-3s. There are divergences that track evolutionary change, certainly, but no specific lineages have been associated with specific functions.

### 14-3-3 PHOSPHORYLATION

Phosphorylation events having a physiologically relevant impact on 14-3-3s have been identified in both plants and animals under certain conditions, but there is not yet a cohesive model for the role phosphorylation plays in defining functional specificity. However, known phosphorylation sites are not universally conserved in 14-3-3s, making phosphorylation an event that could mark certain isoforms for certain functionalities.

A number of phosphorylation events been reported in mammalian 14-3-3s at S58, S64, S132, S185, T233, and Y179 (Aitken et al., 1995; Dubois et al., 1997a,b; Furlanetto et al., 1997; Megidish et al., 1998; Gringhuis et al., 2005). Some of these sites are not conserved across all isoforms allowing potential for specific effects. For example, the S58 phosphorylation site is not conserved in the 14-3-3σ and 14-3-3τ isoforms. 14-3-3β and 14-3-3ζ appear to be the only 14-3-3s phosphorylated at S185 *in vivo* even though two other isoforms contain a serine residue at this site. Furthermore, this phosphorylation event has only been detected in brain and not in other tissues (Aitken et al., 2002).

The first report of phosphorylation of a plant 14-3-3 protein was the demonstration that 14-3-3ω can be phosphorylated *in vitro *(Lu et al., 1994). Since then, a number of large scale phospho-proteomics studies have identified plant 14-3-3 proteins in their screens using mass spectrometry, including gravity responses in Arabidopsis (Barjaktarovic et al., 2009) and seed development in oilseed rape (Agrawal and Thelen, 2006). A recent study in Arabidopsis found evidence for phosphorylation of several isoforms at different sites at the C-terminus of the proteins. This is a region of very low sequence conservation (**Figures 2 and 3**) providing the potential for phosphorylation to affect 14-3-3s in an isoform-specific manner (Sugiyama et al., 2008; Reiland et al., 2009). Three Arabidopsis 14-3-3s (χ, κ, ψ) were identified as being phosphorylated by SnRK2.8 in roots at S93/95 (Shin et al., 2007). This serine residue is conserved across only five of the Arabidopsis isoforms. Tyrosine phosphorylation has also been reported in Maize and Arabidopsis 14-3-3s (Olivari et al., 2000; Giacometti et al., 2004). Few studies have examined the functional effects of these 14-3-3 modifications in plants.

Phosphorylation events that do have a biochemical impact on 14-3-3s have primarily been characterized in mammals. Some of these phosphorylation sites have shown to be conserved in plant 14-3-3s and may play a similar functional role (Aitken et al., 1995; Dubois et al., 1997a,b; Furlanetto et al., 1997; Megidish et al., 1998; Gringhuis et al., 2005). For instance, the phosphorylation of human 14-3-3ϖ at T233 has been shown to modify the ability of 14-3-3s to interact with clients by introducing a conformational change of the C-terminus (Dubois et al., 1997b; Kubala et al., 2004; Obsilova et al., 2004; Clokie et al., 2009). In plants there are three sites within the C-terminal tail (helix 9) which are phosphorylated during seed development in Brassica (Agrawal and Thelen, 2006) and, as mentioned above, there is also evidence for phosphorylation at the C-terminus in Arabidopsis (Sugiyama et al., 2008; Reiland et al., 2009). Based on the data from mammals, it can be predicted that this may affect the conformation of the C-terminus and client binding. Phosphorylation of human S58 affects client binding, and compromises the ability to dimerize (Powell et al., 2003; Woodcock et al., 2003; Gu et al., 2006; Jagemann et al., 2008; Sluchanko et al., 2011; Woodcock et al., 2010). S58 corresponds to residue S62 in Arabidopsis 14-3-3ω. This site is conserved across all Arabidopsis isoforms with the exception of 14-3-3κ and 14-3-3λ. In plants, phosphorylation at Y179 and S185 affects client binding, although the mechanism and their effects on 14-3-3 structure is not known (Tsuruta et al., 2004; Gao et al., 2005; Sunayama et al., 2005; Yoshida et al., 2005; Barry et al., 2009). Potential tyrosine phosphorylation of the 14-3-3s at Y137 decreases binding to the H+-ATPase (Olivari et al., 2000; Giacometti et al., 2004). Although the Y179 and Y137 sites are conserved across all isoforms, S185 is not present in 14-3-3μ.

**FIGURE 4:**
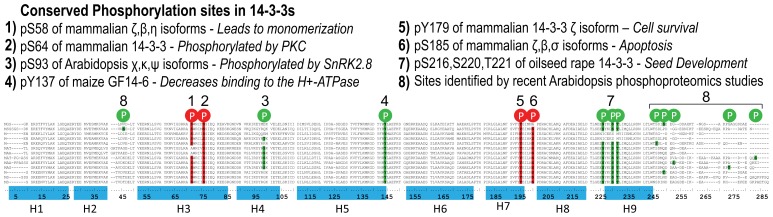
**14-3-3 Phosphorylation sites.** 14-3-3 residues that are phosphorylated in animal or plant 14-3-3s and that are also conserved in Arabidopsis 14-3-3 protein sequences are listed. Sites phosphorylated in mammalian isoforms are indicated as red blocks, and sites phosphorylated in plant isoforms are indicated as green blocks on the alignment of the 13 isoforms. In cases where the upstream kinase or biochemical/functional impact of the phosphorylation is known, this is indicated above the alignment. Isoform order, top to bottom: μ, ι, π, о, ε, κ, λ, χ, φ, ω, ψ, *υ*, υ. The nine alpha-helices of the 14-3-3 secondary structure are indicated. 1. (Megidish et al., 1998); 2. (Dubois et al., 1997a); 3. (Shin et al., 2007); 4. (Giacometti et al., 2004); 5. (Barry et al., 2009); 6. (Aitken, 1995; Tsuruta et al., 2004); 7. (Agrawal and Thelen, 2006); 8. (Reiland et al., 2009; Sugiyama et al., 2008; Mayank et al., 2012).

Since phosphorylation sites are not conserved across all isoforms, differential phosphorylation could lead to increased functional specificity within 14-3-3s (Moreira et al., 2008). This leads to a situation where discussion of 14-3-3s must accommodate and acknowledge not only the primary sequence difference among 14-3-3s but also the differential phosphorylation among 14-3-3s (see **Figure 4**).

### 14-3-3 DIMERIZATION

The 14-3-3 isoforms are present in cells as some combination of homodimers or heterodimers. Dimer formation in 14-3-3s involves interactions within residues of the first four alpha helices (Gardino et al., 2006). In any cell containing multiple isoforms, if all isoforms could dimerize freely with each other, this would allow for many different combinations. It has been argued that this could dilute out any functional differences between or among isoforms (Wilker et al., 2005). However, there is evidence, particularly from the mammalian literature, that fully random dimerization is not always the case and that there are preferences for certain dimer combinations. This potential is provided by the fact that helices 1 through 4 do contain some of the areas of higher sequence variation among isoforms.

It has been suggested that homodimers and heterodimers could have different functions (Aitken, 2002). In mammalian systems, studies have shown that two isoforms in particular, 14-3-3σ and 14-3-3ε, show a preference to form either homodimers or heterodimers. In one study, immunoprecipitation of endogenous 14-3-3σ from a human cancer cell line showed that it was present primarily as homodimers whereas 14-3-3β formed heterodimers to a much greater extent (Wilker et al., 2005). It is thought that this preference of 14-3-3σ to homodimerize allows it to bind isoform-specific clients and play a specific role in the DNA damage checkpoint that is not shared with other isoforms (Wilker et al., 2005). Conversely, 14-3-3ε has been detected only as heterodimers when expressed *in vivo* (Chaudhri et al., 2003; Yang et al., 2006). Studies of the crystal structure of the mammalian isoforms have helped to explain why this preference for homodimers or heterodimers may exist. There are a number of variable amino acids in the dimer interface between isoforms. Subsequently, the number of stabilizing salt bridges in the dimer interface varies between one and three depending on the isoform. In the case of the 14-3-3σ homodimer, there are three salt bridges and also extra interactions between aromatic ring side chains, stabilizing the homodimers (Benzinger et al., 2005; Wilker et al., 2005; Gardino et al., 2006; Yang et al., 2006). If 14-3-3ε was to form a homodimers, there would only be one stabilizing salt bridge and therefore may be the reason why ε prefers to form heterodimers (Gardino et al., 2006; Yang et al., 2006). The two yeast 14-3-3s also show an inclination to form heterodimers rather than homodimers (Chaudhri et al., 2003). There is also evidence for preferences for particular heterodimer partners in mammals. Specificity was shown in the dimer partners co-immunoprecipitated with myc-tagged 14-3-3ε or 14-3-3γ expressed in neuronal cells which did not appear due to the amount of total endogenous isoform present (Chaudhri et al., 2003).

In plant systems, the issues surrounding dimerization are even less resolved, particularly *in vivo*. Our early studies looking at the *in vitro* dimerization potential of different recombinant Arabidopsis 14-3-3s hinted that there may be some differences between isoforms in their preferences for dimer partners. In that study, although all four Arabidopsis isoforms tested were able to form all combinations of both homodimers and heterodimers in yeast-2-hybrid, there were interesting results when different isoform pairs were renatured *in vitro *and analyzed by native gels, which suggested there are differences in the ratios of homodimers to heterodimers present between different isoform pairs (Wu et al., 1997). In another Arabidopsis study, TAP tagged 14-3-3ω expressed in cell suspension cells was detected as both homodimers as well as heterodimers with nine other isoforms suggesting that in cells this isoform is able to form dimer partners with multiple isoforms (Chang et al., 2009). In contrast, in a yeast two-hybrid study with cotton 14-3-3s, none of the six isoforms were able to form homodimers, yet heterodimers were detected. Selectivity was also shown in the particular heterodimer partners of the isoforms (Zhang et al., 2010).

Dimerization between different 14-3-3s is, therefore, likely to be variable and depend at least on the intrinsic affinities among 14-3-3s isoforms. Further, dimerization is likely to be variable within cell types, depending on the expression levels and sub-cellular localization of different isoforms, in addition to the phosphorylation status of isoforms. As mentioned above, phosphorylation of 14-3-3s can inhibit dimerization (Woodcock et al., 2003; Gu et al., 2006). There is evidence in mammals that monomeric 14-3-3s may be functional too and may have different client binding properties to the dimers (Shen et al., 2003; Zhou et al., 2003; Han et al., 2010). This leads to the conclusion that dimerization status, particularly the degree of heterodimerization, must be accounted for in any model of 14-3-3 isoform specificity.

## STATEMENT OF THE ISSUE

Given the clear presence of diversity in the primary structure of 14-3-3s in plants, and further given that this primary sequence diversity can be amplified by phosphorylation and by heterodimerization, what are the biochemical, cellular, and physiological impacts of that diversity? Is the diversity an evolutionary collection of biochemically irrelevant genetic drift or is the diversity a reflection of true functional specification within the 14-3-3 family? With multiple isoforms present in any cell, the potential for dilution through dimerization, and with the common theme of interaction with certain phosphorylated peptides, it is difficult to construct obvious modes of isoform functional specificity.

Our theory, however, is that true cellular and biochemical functional specificity does indeed exist among members of the 14-3-3 family. Disproving specificity as a hypothesis would be difficult, as lack of data cannot constitute proof of the hypothesis as being false. Therefore, our formally stated null hypothesis would be that *no* functional specificity exists among 14-3-3s. Rejection of this null hypothesis would be accomplished by any clear observations of specific function between or among 14-3-3 isoforms, which would then support our broader theory of specificity among 14-3-3s. In general, we posit that certain 14-3-3s in a given cellular context – and only those 14-3-3s – will have specific interactions that are not shared with other 14-3-3s, and as a result, certain 14-3-3s will have functional phenotypes revealed by mutation. Data are emerging from the literature in support of this notion. As a particular outcome for developing tests of the hypothesis, we posit that multiple 14-3-3s may be present in equal concentrations within a cell, yet each may biochemically interact with different clients and each may therefore have different cellular functions and localizations. Data from the literature and some primary data are presented in support of this notion. Full consideration of all factors suggests that it is possible that restricted groups of isoforms may have interactions and functions that are shared within that group. Members of a certain evolutionary branch would be one example of a group with potential shared specificities. 14-3-3s phosphorylated at a certain residue might also constitute a functionally specific group. And it is also possible that some biochemical and cellular functions could be shared by all isoforms. But the main idea of the theory is that out of the family of conserved, dimerizing 14-3-3s within a plant cell, certain isoforms or isoform groups do have certain specific biochemical interactions and functions.

## ELEMENTS OF THE ISSUE

In order to address the theory and set up observational tests of the null hypothesis, we present data of *functional redundancy* that would *fail to reject* the null hypothesis, and also present observations of *specificity* that appear to *reject* the null hypothesis, thereby supporting the theory of specificity. Note that the theory of specificity does not explicitly deal with cell-specific expression of 14-3-3s, though this is an important overall component of 14-3-3 roles. It is certainly possible that developmental specificity of expression could present certain 14-3-3s with prominent roles in certain tissues, regardless of the specificity at the cellular or biochemical level. We therefore discuss briefly just how much tissue-specific expression of 14-3-3s might exist, then we examine redundancy and specificity at the cellular and biochemical levels as these levels do address directly the questions of functional specificity among protein isoforms.

### EXPRESSION SPECIFICITY

In mammals, early 14-3-3 literature reported differences in the distribution of isoform transcripts in different regions of the brain by in situ hybridization, and also in other mammalian tissues by northern blot analysis (Watanabe et al., 1993, 1994). The difference in expression between brain structures is also supported by data from microarray studies in mice which show14-3-3γ to have by far the highest expression of any isoform in hippocampus. Conversely, the hypothalamus shows very low 14-3-3γ expression compared to the other isoforms, with 14-3-3β showing the highest expression levels followed by 14-3-3θ/τ (Rangel et al., 2009; Kim, 2012; Nijnik et al., 2012; Wefers et al., 2012). 14-3-3σ in particular shows notable differences in tissue expression compared to the other isoforms as it has only been detected in epithelial cells (Leffers et al., 1993). 14-3-3σ has received significant attention due to its proposed role as a tumor suppressor and thought to be linked to the exit of cells from the stem cell compartment of epithelia to become differentiated (Hermeking, 2003). As described in the dimerization section above, this specialized role may be due to its unique structural characteristics, not shared with other isoforms.

**FIGURE 5:**
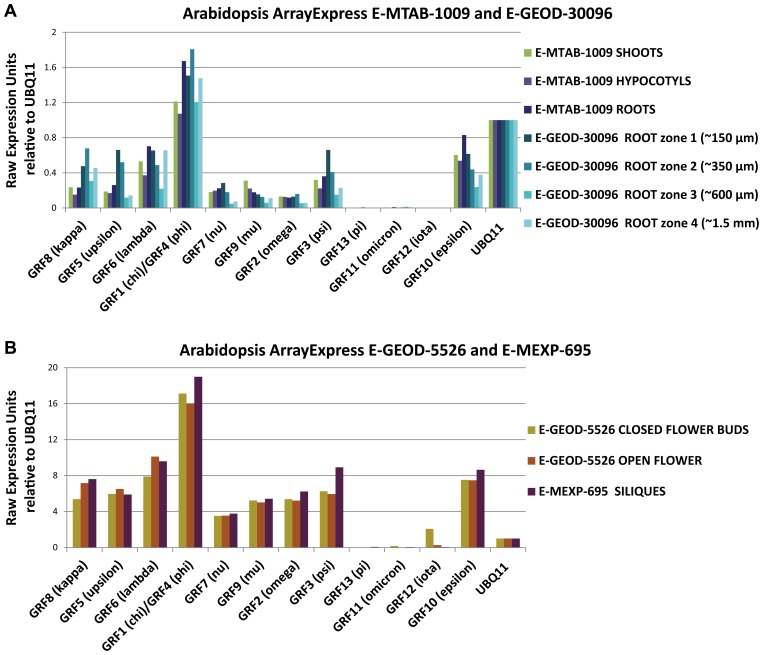
**Representative charts showing similarity in the 14-3-3 isoform genes expression pattern across different plant tissue in Arabidopsis thaliana based on selected microarrays expression data from ArrayExpress database.** The experiment IDs in ArrayExpress database are provided in the chart title. Gene expression data was analyzed for only untreated, wt, Colombia cultivar plants. *Y* axis raw expression units relative to UBQ11 (At4g05050). Data from E-MTAB-1009 and E-GEOD-30096 was used to plot 14-3-3 isoform genes expression level across whole shoots, hypocotyls, and roots as well as root zones. The distance in the legend defines the distance from the root tip **(A)**. The two independent experiments were analyzed, E-GEOD-5526 and E-MEXP-695 to plot 14-3-3 isoform genes expression level across reproductive tissues: siliques, closed flower buds, and open flowers were included. The asterisk indicates the higher 14-3-3 iota isoform expression level exclusively in closed flower buds **(B)**. Notes: GRF (λ)/GRF4 (φ) label: GRF1 (λ alias GF14 λ; At4g09000), and GRF4 (φ alias GF14 φ; At1g35160) share same probe 255079_s_at on A-AFFY-2 - AffymetrixGeneChip Arabidopsis Genome [ATH1-121501]. As other _s_at probes (_s indicating Similarity) this probe represents a set with all probes common among multiple transcripts within a gene family, in this particular case chi and phi therefore both genes GF14 PHI and CHI could be found as annotated for the same probe. All other 14-3-3 members have unique probe IDs on ATH1-121501 arrays (_at individual probe).

In plants, early reports showed that Arabidopsis 14-3-3χ is differentially expressed in tissues by analysis of promoter activity and in situ hybridization (Daugherty et al., 1996). 14-3-3ω shows relatively high expression in flowers compared to leaves and stems by semi quantitative RT-PCR analysis, in contrast to 14-3-3κ and 14-3-3λ which show fairly even expression across these tissues (Sorrell et al., 2003). One isoform in particular, 14-3-3ι, shows a distribution more restricted to flowers hinting that it may play a specialized role here (Rosenquist et al., 2001). This is supported by microarray data from a number of studies which shows the 13 isoforms show a fairly similar expression pattern across tissues (Dinneny et al., 2008; Griffiths et al., 2011), with the exception of 14-3-3ι where transcripts are almost completely restricted to closed flower buds (Hennig et al., 2004; **Figure 5**). It should be noted that in situ hybridization and promoter fusion studies give an impression of greater cell specificity than observed by large scale microarray studies of tissues or organs.

Rice 14-3-3 isoforms shows differences in expression across tissues and this pattern is different between isoforms (Chen et al., 2006; Yao et al., 2007; Purwestri et al., 2009). Interestingly, in one study the Os14-3-3-C isoform, which showed the most evenly high expression across all tissues studied, was also the most successful isoform in complementing deletion mutants of the 14-3-3 homologues in yeast (see below). This led the authors to speculate that isoforms such as Os14-3-3-C, which show ubiquitous expression across all tissues, may play very general and fundamental roles in rice. On the other hand, isoforms like Os14-3-3-G, which are unable to rescue the yeast 14-3-3 knockout, and which shows a more restricted tissue distribution, may play unique roles in response to specific conditions or developmental stages (Yao et al., 2007). A similar variation in tissue distribution between isoforms exists in cotton (Shi et al., 2007), soybean (Zhang et al., 2010; Li and Dhaubhadel, 2011), and broad bean (Emi et al., 2001). Vf14-3-3a shows higher expression in guard cells compared to the three other isoforms. It was proposed therefore that Vf14-3-3a may have a higher affinity for binding to the H^+^-ATPase than Vf14-3-3b, another isoform that is expressed in guard cells (Emi et al., 2001).

### EVIDENCE FOR REDUNDANCY

Redundancy in 14-3-3 function can be examined from several perspectives. One perspective is physiological and can be approached by experiments with genetic mutations or TDNA knockouts in plants. The other perspective is biochemical, which can be examined by assays of direct biochemical interaction *in vitro*.

#### Physiological redundancy

One key observation in support of redundancy would be the lack of observable phenotype in a 14-3-3 TDNA knockout. In Arabidopsis, early studies indicated that several different 14-3-3 TDNA insertion mutants had no observable phenotype at all. Further, several combinations of 14-3-3 TDNA insertions apparently had no observable phenotype (Krysan et al., 1996, 1999), allowing for the continued debate over the extent of 14-3-3 functional redundancy in plants (Comparot et al., 2003; Ferl, 2004; Chevalier et al., 2009).

There are also examples of different isoforms being involved with the same physiological response. Several 14-3-3s are involved in drought-related processes, and specific 14-3-3 isoforms have been implicated at the protein level in drought-stress, where overexpression of different single isoforms, At14-3-3λ or ZmGF14-6, can enhance drought tolerance (Yan et al., 2004; Campo et al., 2012). In the yeast *Saccharomyces cerevisiae*, a knockout of one of the two 14-3-3 forms results in a viable line, while the double knockout is lethal, suggesting the two isoforms found in yeast are functionally redundant (Lottersberger et al., 2006, 2007; Zannis-Hadjopoulos et al., 2008). This simple observation strongly suggests that the yeast 14-3-3s are essentially redundant, even though the single mutants do exhibit some growth differentials. The yeast double 14-3-3 mutant can be rescued by four Arabidopsis isoforms (van Heusden et al., 1996; Sorrell et al., 2003) and a human isoform (Zannis-Hadjopoulos et al., 2008). This is a profound observation that suggests strongly that most, if not all, of the 14-3-3 interactions that are required in yeast are retained in the isoforms of Arabidopsis and humans. However, recent results suggest that this conclusion is not universal. The Os14-3-3-C isoform is much more successful in complementing yeast deletion mutant than Os14-3-3-G (Yao et al., 2007). While there may be basic or fundamental activities in yeast that can be supported by many isoforms from plants, it may not be universally true that all plant isoforms can complement yeast.

#### Biochemical redundancy

At the biochemical level there is evidence that 14-3-3 isoforms have overlapping client binding profiles. Multiple mammalian isoforms show a very similar sequence binding preference based on screens across peptide libraries (Yaffe et al., 1997; Muslin et al., 1996). Six mammalian isoforms can all bind Bad *in vitro* and are able to reduce the apoptotic effects of Bad on cells (Subramanian et al., 2001). In plants studies, three different Arabidopsis isoforms were able to activate the H-ATPase (Baunsgaard et al., 1998) and surface plasmon resonance has shown that three barley isoforms can bind a lipoxygenase enzyme with similar affinities (Holtman et al., 2000). While not exhaustive in their treatments across isoforms, these studies and other studies indicate that at the biochemical interaction level, there can be overlap and similarity among isoforms with regard to client affinities.

### EVIDENCE FOR FUNCTIONAL SPECIFICITY AMONG ISOFORMS

Although data supporting 14-3-3 functional specificity in plants are few, *any* observed specificity for any 14-3-3 function would reject the formal null hypothesis of complete isoform redundancy. From the literature there are now several cases of individual 14-3-3 TDNA insertions conditioning an observable phenotype. The fact that any phenotype can be assigned to 14-3-3s argues for specificity from first principles. Given the wide distribution of 14-3-3 isoform expression (**Figure 5**) it is likely that most cells have 14-3-3s present in addition to the one that is knocked out, hence phenotypes for individual 14-3-3s argues for specificity at the cellular level. Moreover, there are also now a few comparative studies in which phenotypes are known to be conditioned by one 14-3-3 isoform but not another.

#### Functional specificity revealed by TDNA mutations

Functional diversity of plant 14-3-3s is widely documented, and includes primary metabolism, abiotic and biotic stress, light-mediated processes, hormonal signaling, and basic cell growth and division (reviewed in Aitken, 2002; Denison et al., 2011). However, discrete examples of isoform functional specificity revealed by individual TDNA mutations are now emerging.

The Arabidopsis 14-3-3 isoforms υ and μ are involved in light sensing and signaling (Mayfield et al., 2007). Mutant υ and μ plants flower later and exhibit long hypocotyls under red light, with little effect under blue or far-red light. The long hypocotyl phenotype is consistent with a role for 14-3-3υ and μ in phytochrome B signaling. Yeast two-hybrid and co-immunoprecipitation assays indicate that 14-3-3υ and μ proteins physically interact with CONSTANS, a central regulator of the photoperiod pathway. Together, these data indicate a potential role for these specific 14-3-3 isoforms in affecting photoperiodic flowering via interaction with CONSTANS, possibly as integrators of light signals sensed through the phytochrome system. These observations set at least 14-3-3μ and 14-3-3υ apart as 14-3-3s individually capable of regulating of flowering time.

However, even though both 14-3-3μ and *υ* affect flowering time, they are not themselves redundant with each other. In the first place, redundancy between 14-3-3μ and *υ* would preclude either one from having a flowering phenotype as an individual TDNA mutation. In addition, 14-3-3μ TDNA insertion mutants have a distinctive short root phenotype, a phenotype that is not present in 14-3-3υ TDNA insertions (Mayfield et al., 2012). This observation suggests that functional specificity is likely context dependent.

In a demonstration of remarkable specificity that is likely dependent on cellular context, the Arabidopsis κ and λ isoforms differentially affect Phot2 signaling in guard cells. κ and λ are closely related isoforms on a well-supported, separate evolutionary branch within the non-ε group. κ and λ differ by only a few amino acids in primary sequence, yet λ is required for normal stomatal opening by Phot2 while κ insertion mutations have no effect (Tseng et al., 2012). This observation suggests that in certain very specialized cell types, in this case the guard cells of the stomata, very specific requirements for certain specific 14-3-3 do exist (see also below).

#### Biochemical specificity

Plants have shown variation among isoforms in their affinity for specific client proteins. In Arabidopsis, recombinant constructs of 14-3-3 ω, χ, υ, φ, and ψ were comparatively tested for their ability to interact with phosphorylated nitrate reductase from spinach leaves, demonstrating drastically different levels of interaction (Bachmann et al., 1996). Different levels of biochemical inhibition of nitrate reductase have also been shown (Lambeck et al., 2010, 2012). Recombinant 14-3-3s have different affinities for H-ATPase (Rosenquist et al., 2000) and those affinities are not in the same relative order as described for nitrate reductase interactions. In addition, yeast two-hybrid studies in barley show differences in interaction levels for clients (Bornke, 2005; Schoonheim et al., 2007) and barley isoforms differ in their ability to inhibit NR or regulate K^+^ channels (Sinnige et al., 2005a,b). Six cotton 14-3-3s showed specificity in target binding in yeast two-hybrid study (Zhang et al., 2010). These studies do not necessarily stand in contrast to those studies cited above in which biochemical redundancy was demonstrated. Taken together, it is likely that there is a range of biochemical interaction affinities among isoforms, with isoforms demonstrating similar affinities for certain peptides or protein clients while also demonstrating specific affinities for some peptides and protein clients.

#### Subcellular localization as a measure of specificity

Subcellular localization can be a powerful indicator of 14-3-3 specificity among isoforms. The localization of a given 14-3-3 could be viewed as being directed by two forces. One force is the intrinsic localization of the individual 14-3-3 itself due to signals present in the 14-3-3 sequence. 14-3-3s are known, for example, to contain a Nuclear Export Signal (NES), and that signal is thought to aid in the 14-3-3-mediated movement of transcription factors out of the nucleus (Graves et al., 2001; Brunet et al., 2002; Zhao et al., 2004; Nishino et al., 2008). The other force influencing 14-3-3 localization would be the location of the client protein. Localization of the 14-3-3/client complex would be dependent upon not only the localization signals of the 14-3-3 but also on the localization signal of the client protein. If the 14-3-3 NES was the dominant localization force, 14-3-3s would be expected to be prominently cytosolic in their localization. To expand this discussion we consider two possible 14-3-3 localization notions.

**FIGURE 6:**
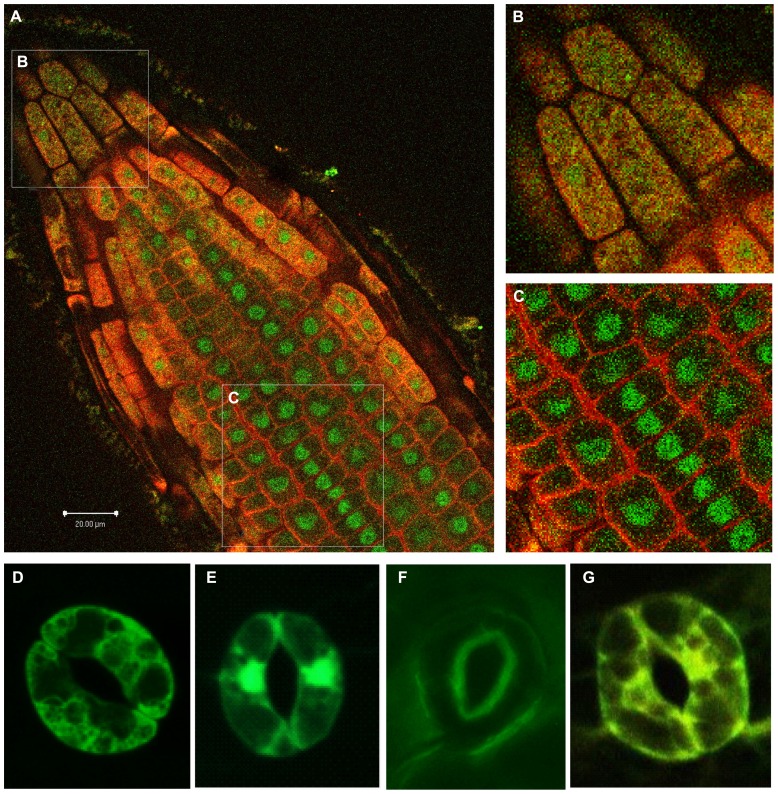
**Distinctly different 14-3-3 localizations determined by confocal microscopy of Arabidopsis.** Isoform-specific antibodies that have been directly labeled with either Alexa-488 or Alexa-568 and used for *in situ* labeling. 14-3-3 λ (green) predominates in the nucleus while 14-3-3 ε (red) predominates in the cytoplasm and plasma membrane. A single longitudinal optical slice of a root tip is shown in **(A)**. **(B,C)** Provide closer views of the boxed sections of the root in **(A)**. The root tip **(B)** exhibits an even distribution of 14-3-3-λ and 14-3-3-ε throughout the cells, and a section father back in the root **(C)** exhibits a clear partitioning of 14-3-3-λ into the nucleus and 14-3-3-ε to the plasma membrane/cell wall. Guard cell subcellular localization of four GFP-tagged 14-3-3 isomers is shown in the bottom row: ε **(D)**, κ **(E)**, λ **(F)**, and φ **(G)**.

At one level, consider the number of potential interactions for a single given isoform. That isoform would have only one set of intrinsic signals, an intrinsic localization based on its own structure. The other forces determining localization of the 14-3-3 would be derived from its interacting client protein. Most estimates of potential 14-3-3 interacting proteins in any cell, based on the presence of the canonical 14-3-3 recognition sequences, range from hundreds to thousands (Sehnke et al., 2002a). If these interacting proteins actually occur and are distributed randomly among cell compartments (nucleus, cytoplasm, and plasma membrane, for example), the given 14-3-3 isoform should be broadly dispersed among cellular locations because of the many locations of its clients. Under such conditions of promiscuous client interaction, a specific subcellular localization of any given 14-3-3 would be very unlikely. However, there certainly are distinct 14-3-3 subcellular localizations that are tissue-specific and different among 14-3-3 isoforms in plants (Sehnke et al., 2002a; Maraschin et al., 2003; Paul et al., 2005; Campo et al., 2012). Further, disrupting the client interactions can influence the localization of specific 14-3-3s (Paul et al., 2005; Sabina et al., 2007). Thus any observable, limited, specific subcellular localization of a 14-3-3 supports the notion that the 14-3-3 is interacting with a specific subset of cellular proteins (see **Figure 6**).

At a second level, consider the potential interactions of two different 14-3-3 isoforms present in the same cell. If the two isoforms were interacting with the same clients, the two isoforms would be present in the same subcellular locales. However, isoform-specific immunocytochemistry demonstrates that individual 14-3-3s can and do partition themselves in different locations within the cell. **Figure 6** illustrates the localization of 14-3-3χ and 14-3-3ε in a young Arabidopsis root. Both χ and ε are robustly expressed in roots (see **Figure 5**). Yet the localization patterns for χ and ε are distinctly different across the cells of the root tip (**Figure 6A**). In the central cells marked with the C box in **Figure 6**, χ is largely nuclear, while ε is largely associated with the plasma membrane or cell wall. This single observation has dramatic implications for the notion of isoform-specific functionality. Because χ and ε are in two separate and distinct locations, they cannot be extensively heterodimerizing with each other. If expressed equally and randomly dimerizing, χ and ε would be present in a ratio of 1:2:1 of χ homodimers:χ/ε heterdomers:ε homodimers. Therefore, two-third of the red and green signals would be combined into a yellow heterodimer signal. This is clearly not the case. In fact the majority of signal in these cells is distinctly green or red. Because χ and ε are in separate subcellular locales, they cannot be interacting with the same clients or subsets of clients unless the intrinsic localization signals of the 14-3-3s are distinct and direct the localization of the complex. Moreover, the fact of specific and limited localization of these isoforms suggests that in these cells, χ and ε are interacting with few, potentially unique, client proteins.

In the root tip cells marked with the B box in **Figure 6**, χ and ε are almost evenly distributed with regard to subcellular localization. In these cells they could be heterodimerizing or they could be interacting with a large number of different clients individually. Regardless, this distribution suggests that at least these two isoforms have distinct roles in metabolic processes in the different root zones, and further, that those roles can change depending on specific cell function.

Other specialized cell types exhibit isoform-specific distributions as well. Subcellular localization of specific 14-3-3s was also examined in plants transformed with GFP fusions of individual 14-3-3 isoforms. Subcellular differences among the GFP fusion lines were most apparent in specialized cell types, such as trichomes and guard cells (Paul et al., 2005). The guard cells provide a particularly striking example. 14-3-3ε-GFP is distributed throughout the cytoplasm, but diminished in the nucleus (**Figure 6D**). On the other hand, 14-3-3κ-GFP is almost exclusively nuclear (**Figure 6E**). 14-3-3λ-GFP is generally expressed at lower levels, but when it is found in guard cells, it is always localized to the edges that define the stomata (**Figure 6F**). This marked difference in the distribution of κ and λ was surprising given the similarity of the two isoforms, however, distinct functional roles for these two related isoforms has also been demonstrated with respect to Phot2-mediated processes, where it was shown that λ played a role in stomatal opening in response to blue light, but κ did not (Tseng et al., 2012). The distribution of 14-3-3φ-GFP is seen throughout the cell and nucleus, but is especially prominent along the cytoskeleton (**Figure 6G**). In fact, live imaging with confocal microscopy shows the GFP-tagged φ isoform moving rapidly along the fibers of the matrix, to and from the nucleus (Paul et al., 2005).

### MODEL AND THEORY FOR SPECIFICITY

Data strongly support the theory of specificity in 14-3-3 functional interactions at the cellular level. Two major types of observations cleanly reject the null hypothesis that no specificity exists and that all 14-3-3s are functionally redundant; the presence of phenotypes for 14-3-3 insertion mutants and the differential subcellular localization of isoforms within a cell. Given the wide expression profiles of most plant 14-3-3 genes, it is likely that almost every cell in the plant has a fairly complex population of different 14-3-3 isoforms and that many cells contain many 14-3-3 isoforms. Therefore, the existence of phenotype(s) associated with individual 14-3-3 insertion mutations is strong evidence that the particular isoform plays an important role that is not entirely complemented by other 14-3-3s present in a cell. Earlier, general studies showing that some 14-3-3 insertion mutations displayed no phenotype are now being supplanted by specific studies of well characterized insertion mutations that do display phenotypes. Furthermore, cell biology observations define another element of specificity. Within a particular cell, different 14-3-3s can be found in distinct subcellular locations, demonstrating clearly that the different 14-3-3s cannot be interacting with all of the same clients and serving the same cellular function. Among many, the example of 14-3-3λ and 14-3-3κ is quite compelling and complete. These isoforms are closely related yet show differential localization and differential physiological function within the light signaling pathways of guard cells (Paul et al., 2005; Tseng et al., 2012).

The statement that there can be specificity in 14-3-3 function within a cell is, however, a simplification of a larger and more complex model for 14-3-3 interactions and functions. A full model for 14-3-3s interactions must incorporate a quantitative appreciation for (1) the number of different 14-3-3s present in the cell of interest, (2) the concentrations of each isoform, (3) the heterodimerization affinity among the isoforms present, (4) the affinities for each of the isoforms for each client target protein in the cell, and (5) any modifications of the 14-3-3s. Given these complexities, it is perhaps remarkable that specificity does exist in 14-3-3 function. Redundancies in function also exist. Therefore, a complete model of 14-3-3 roles must also accommodate some pool of overlapping specificities and perhaps some completely redundant functions among isoforms.

The 14-3-3 family of isoforms, dimers, and modified forms should be considered a complex and dynamic system, a system that should be addressed and better understood with each experiment. This will require comparative studies. Where possible, observations about a given 14-3-3 isoform should be compared and contrasted with other isoforms within the same experiment. This is a key element often missing in current 14-3-3 literature. With increasing TDNA mutations publically available, for example, contrasts in experiments should accompany statements of 14-3-3 roles, including data where no phenotype is observed in some mutation(s). Care should be taken to be specific in the description of isoforms in an experiment and conclusions drawn from one isoform should not be considered to be true for other isoforms until tested. The limits of reagents and databases should be taken into consideration. For example, the Affymetrix array does not list a probeset for 14-3-3φ, and the probeset for 14-3-3χ very likely hybridizes with both 14-3-3φ and χ, presenting a likely over estimate of 14-3-3χ expression. Similarly, proteomics peptide databases do not always properly distinguish among isoforms, and peptides can be identified being from a particular isoform when in fact that peptide can be from all members of an evolutionary group. And TDNA insertions can result in various levels of 14-3-3 expression at the protein level. Deeper understanding of the 14-3-3 families and their roles as individual isoforms will therefore require experiments that are comparative among isoforms and a refined approach that increases the ability to identify individual isoforms and isoform groups as experimental contrasts.

The theory of 14-3-3 specificity predicts that, when properly monitored and experimentally contrasted, certain 14-3-3 isoforms have a function that is not present in other isoforms – even if that isoform is present in the same cell. This theory does not preclude that there will be examples of overlapping function, but it does state that some functions will be clearly distinct among isoforms. Other systems of isoform families have embraced this notion. For instance, mammalian Akt protein kinases are highly conserved and function in the regulation of a diversity of cellular functions. Yet phenotypic studies with knockout mice have demonstrated that the three isoforms that comprise this family play distinct roles in the regulation of cellular growth, glucose homeostasis, and neuronal development. This reverse genetics approach establishes that the functions of each Akt isoform do not wholly overlap, and demonstrates that isoform-specific signaling contributes to the diversity of Akt activities in mice (Gonzalez and McGraw, 2009). In *Drosophila*, the demonstration that lines deficient in one or the other of their two 14-3-3 genes were not fully functional lead to the discovery that D14-3-3ε plays a specific role in pole cell migration, a specific function that could not be rescued by 14-3-3 Leo (Tsigkari et al., 2012). Thus it is apparent that specificity in isoform families can be discovered and appreciated within an enabled and aware experimental context.

## Conflict of Interest Statement

The authors declare that the research was conducted in the absence of any commercial or financial relationships that could be construed as a potential conflict of interest.
